# A Pilot Study for Estimating the Cardiopulmonary Signals of Diverse Exotic Animals Using a Digital Camera

**DOI:** 10.3390/s19245445

**Published:** 2019-12-10

**Authors:** Ali Al-Naji, Yiting Tao, Ian Smith, Javaan Chahl

**Affiliations:** 1 Electrical Engineering Technical College, Middle Technical University, Baghdad 1022, Iraq; 2 School of Engineering, University of South Australia, Mawson Lakes SA 5095, Australia; Yiting.Tao@mymail.unisa.edu.au (Y.T.); Javaan.Chahl@unisa.edu.au (J.C.); 3 Zoos South Australia, Adelaide SA 5000, Australia; ismith@zoossa.com.au; 4 School of Animal and Veterinary Sciences, University of Adelaide, Roseworthy SA 5371, Australia; 5 Joint and Operations Analysis Division, Defence Science and Technology Group, Melbourne VIC 3207, Australia

**Keywords:** cardiopulmonary signal, animal health, veterinary, non-contact, vital signs, wildlife, conservation, denoising, wavelet

## Abstract

Monitoring the cardiopulmonary signal of animals is a challenge for veterinarians in conditions when contact with a conscious animal is inconvenient, difficult, damaging, distressing or dangerous to personnel or the animal subject. In this pilot study, we demonstrate a computer vision-based system and use examples of exotic, untamed species to demonstrate this means to extract the cardiopulmonary signal. Subject animals included the following species: Giant panda (Ailuropoda melanoleuca), African lions (Panthera leo), Sumatran tiger (Panthera tigris sumatrae), koala (Phascolarctos cinereus), red kangaroo (Macropus rufus), alpaca (Vicugna pacos), little blue penguin (Eudyptula minor), Sumatran orangutan (Pongo abelii) and Hamadryas baboon (Papio hamadryas). The study was done without need for restriction, fixation, contact or disruption of the daily routine of the subjects. The pilot system extracts the signal from the abdominal-thoracic region, where cardiopulmonary activity is most likely to be visible using image sequences captured by a digital camera. The results show motion on the body surface of the subjects that is characteristic of cardiopulmonary activity and is likely to be useful to estimate physiological parameters (pulse rate and breathing rate) of animals without any physical contact. The results of the study suggest that a fully controlled study against conventional physiological monitoring equipment is ethically warranted, which may lead to a novel approach to non-contact physiological monitoring and remotely sensed health assessment of animals. The method shows promise for applications in veterinary practice, conservation and game management, animal welfare and zoological and behavioral studies.

## 1. Introduction

Monitoring cardiopulmonary activity constitutes a fundamental component of animal care, since the data obtained can detect and be highly predictive of dynamic clinical conditions. Current remote-measurement techniques for monitoring animal physiology such as telemetry require invasive equipment through the use of expensive equipment implanted or affixed through potentially risky physical interventions [1]. Capture and restraint of an animal may induce stress and long-term trauma that also affect the physiological states being measured in both the immediate and longer term [2,3,4,5,6,7].

In addition, handling animals, and placing them in communal clinical facilities may increase the risk of spreading infectious diseases between humans and animals or between animal patients [4,8]. Many species are potentially dangerous due to their size, or their propensity to display innate defensive/aggressive behaviors and fight/flight reflexes; this includes several of the species involved in this study. In a zoological setting these innate dangerous behaviors are compounded by the confines of the facilities, while in their natural habitat, these species are likely to be extremely difficult to approach or capture. It is also dangerous to measure physiological parameters of some wild animals using conventional measurement systems because of their indocility [9,10]. Consequently, non-contact systems for tracking animal cardiopulmonary signals have attracted much attention.

In recent years, non-invasive and non-restrictive sensing techniques have been developed for monitoring animal physiological parameters. For example, Matsui et al. [11,12] proposed a non-contact cardiopulmonary monitoring system based on a microwave radar antenna with a frequency of 1215 MHz to measure the pulse rate (PR) and breathing rate (BR) of rabbits. They reported that their proposed system could also be applied to human subjects behind barriers or under disaster rubble or under biochemical hazard conditions. Another study by Suzuki et al. [13] proposed a respiratory monitoring system based on a microwave radar antenna with a frequency of 10 GHz to measure BR of a Japanese black bear during the hibernation period without any physical contact. A study by Zeng et al. [14] constructed a non-contact monitoring system to extract respiratory activity of rats using the signals acquired from apparent respiratory movements based on a Doppler radar antenna operating at 5.8 GHz. Huang et al. [15] used a 60-GHz Doppler radar to extract the cardiopulmonary signal of an anesthetized rat even when respiratory movements were invisible to an observer. Although Doppler radar is a competitive candidate for veterinary sciences and zoology applications, it requires specialized hardware and is unsuitable for long-term monitoring since it is not selective for subject position or multiple subjects. In addition, it is prone to noise and motion artifacts resulting from subject movement and small Doppler shifts [16,17]. A recent study by Noble et al. [1] proposed a novel, affordable, non-contact monitoring system based on an electric field sensor to record cardiopulmonary signal and stereotyped motor behaviors of rodents in their domestic cage environment. However, the sensor used in their study was limited to short distances within the electrical field and may require additional costs for modifications to the environment, for example shielding the cage to isolate the signal of interest [1].

Thermal cameras have also been intensively used in animal research [18,19,20,21,22,23,24,25,26] because they are suitable for long-term monitoring and dark environments. However, limitations for such measurements include difficulty in extracting the signal with a partially occluded region of interest (ROI), ambient environmental thermal noise, high cost, and they are limited to comparatively short distances due to limitations of resolution and optics, size, cost which are all connected to thermal imaging physics and lack of a consumer market for the devices [16,17].

Digital visible light cameras on the other hand may be more appropriate for animal monitoring for a number of reasons. Digital visible light cameras provide at least three visible channels with high resolution, intensity (number of bits per pixel) and both spatial (number of pixels per degree) and temporal (number of frames per second) capacity, all due to market demand. Furthermore, flexibility with visible light optical design, offering panoramic, microscopic and telescopic solutions in well-integrated commercial product families, allows capturing videos at many scenarios. Tailored fields of view allow processing of multiple ROIs in parallel, or in series based on availability. The mass market has led to low cost [16,17] and affordable optics that can be used to extract signals from long distances relevant to both surveillance and conservation applications [27]. A study by Zhao et al. [28] used a video camera as a non-contact sensor to extract cardiopulmonary signals from both humans and animals (fish, mouse and pig). Their study relied on monitoring dynamic variations of physiological parameters, including PR and BR. The first dynamic variation measured was the phenomenom that results from the variations of blood volume during the cardiac cycle that changes the amount of light absorbed by the blood vessel. The second dynamic variation was that resulting from respiration-induced movement that leads to cyclic change in the amount and direction of reflected light from the skin. Both PR and BR were measured from single color channel images captured by a video camera based on image intensity analysis. However, their system would only work when the subjects were in front of the camera with a clear ROI, and thus would probably be unsuitable for unrestrained subjects. Another study by Blanik et al. [29] proposed imaging photoplethysmography (iPPG) (the variations in the optical properties of the skin tissue related to cardiopulmonary activity) using a video camera for monitoring cardiopulmonary activity of anesthetized pigs in a clinical environment. Limitations included the need to anesthetize the animals before the experiment and some restrictions concerning the measurement environment (e.g., illumination conditions) [29]. Recently, Unakafov et al. [30] proposed a non-contact pulse-monitoring system to extract the iPPG signal from a red, green and blue (RGB) facial video of rhesus monkeys. Although iPPG is a promising technology for non-invasive and non-contact remote estimation of cardiopulmonary signals in non-human primates, application of the technique to animals with hair-covered skin, feathers or thicker epidermal layers would be limited.

In the current pilot study, a new, non-contact, non-invasive and cost-effective monitoring system based on digital camera imagery was explored to extract cardiopulmonary signal (PR and BR) of unrestrained animals at different distances using motion on the animal body surface caused by cardiopulmonary activity. This technique contributes to extraction of the cardiopulmonary signal for animals with these more difficult surface textures, since the technique relies on motion more than the skin color variations on which iPPG technology depends. Use of image motion and signal processing also allows the technique to extract the cardiopulmonary signal without a need to anesthetize the animals for specific reasons that will become clear in the derivation and results.

This paper is organized as follows: Section 2 presents the methods and procedures of the proposed imaging system, including the animals and ethical considerations, data acquisition and the experimental setup, system framework and data analysis, and the MATLAB graphical user interface. The experimental results of the proposed imaging system with different animals are presented and discussed in Section 3 and Section 4, respectively. Finally, concluding remarks are outlined in Section 5.

## 2. Methods and Procedures

### 2.1. Animals and Ethical Considerations

The research presented in this pilot study encompassed 10 species of zoo animals including, a giant panda (Ailuropoda melanoleuca), African lions (Panthera leo), a Sumatran tiger (Panthera tigris sumatrae), a koala (Phascolarctos cinereus), a red kangaroo (Macropus rufus), an alpaca (Vicugna pacos), a little blue penguin (Eudyptula minor), a Sumatran orangutan (Pongo abelii) and a Hamadryas baboon (Papio hamadryas). All experiments were reviewed and approved by the Animal Ethics Committee (AEC) of the University of South Australia (protocol number: U06–18). The experiments complied with the National Health and Medical Research Council (Australia) code for the care and use of animals in experimental research. This pilot research was required to be based only on filming is such a way that the animals’ routine was not disrupted. No animals had known health problems at the time of filming.

### 2.2. Data Acquisition and Experimental Setup

**Figure 1:**
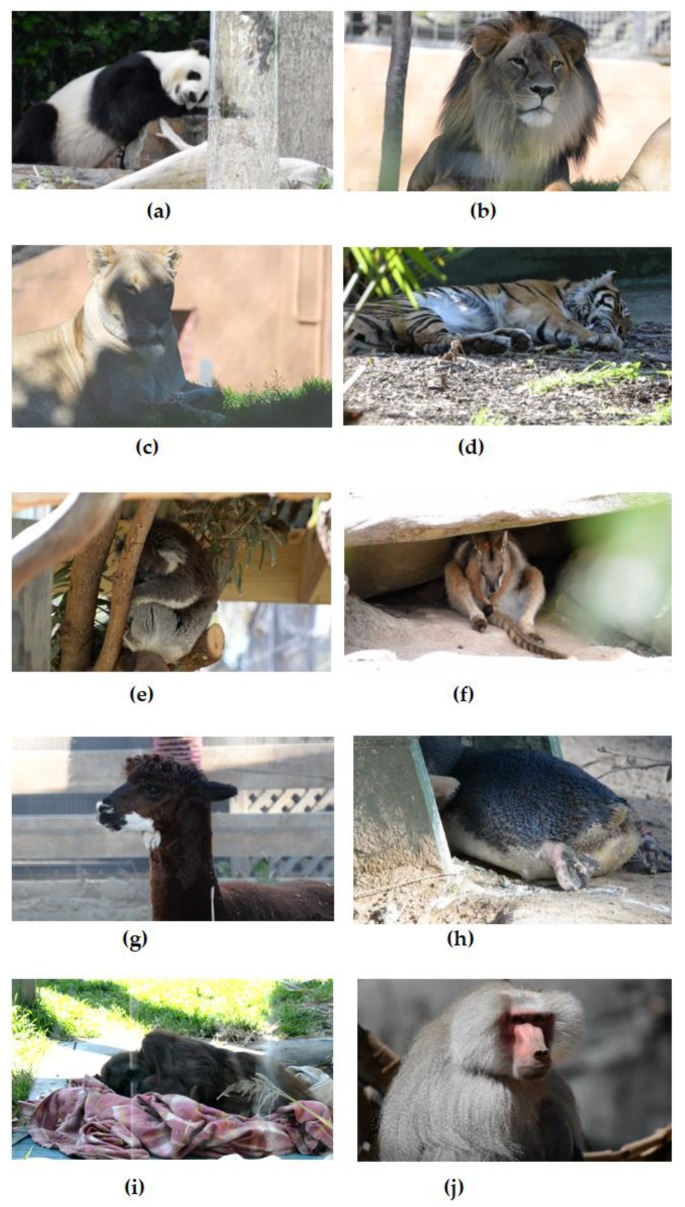
Data collection from 10 zoo animals, (**a**) giant panda, (**b**) African lion, (**c**) African lioness, (**d**) Sumatran tiger, (**e**) koala, (**f**) red kangaroo, (**g**) alpaca, (**h**) penguin, (**i**) Sumatran orangutan, and (**j**) Hamadryas baboon.

Data was collected during two days in the Adelaide Zoo located in Adelaide, South Australia [31]. A digital camera (Nikon D610, Nikon Inc., Tokyo, Japan) was installed on a tripod at a distance approximately of 3 to 40 m from the animals. The distance relies on the animal kinds and how dangerous it is, size of the cage and the safety distance. The video data was captured using a 200–500 mm lens (Nikon AF-S NIKKOR 200–500mm f/5.6E ED VR, Nikon Inc., Tokyo, Japan) with a resolution of 1920 × 1080 and a frame rate of 30 fps, saved in MOV (A QuickTime video file extension) on the laptop. The videos were acquired under ambient lighting conditions for 1–3 min. for each animal and repeated at different times to obtain sufficient video data for experimental purposes. However, we only cut one minute from each video when the animal moved the least to remove any motion artefacts that were not related to internal processes. A single frame from the data collection from the 10 species of zoo animals is shown in Figure 1. As a pilot study instrumenting the animals for experimental control purposes was not justifiable or practical due to the early stage of the research and the risk to all involved, and this is planned as a subsequent experiment.

### 2.3. System Framework and Data Analysis

**Figure 2:**
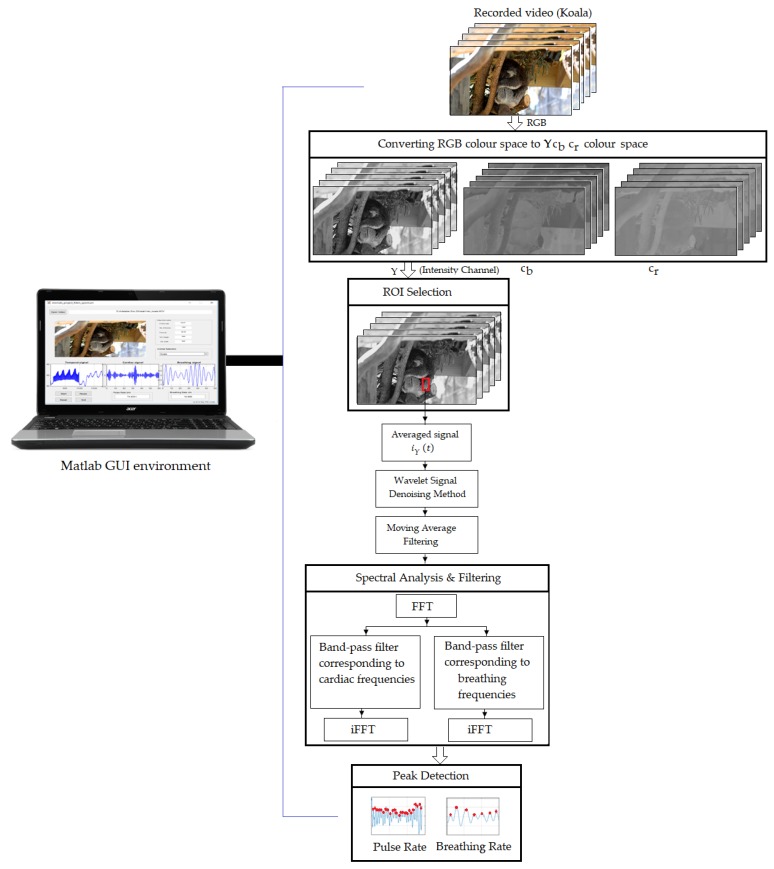
Schematic diagram illustrating the process by which non-contact video data obtained were acquired using a digital camera to extract the cardiopulmonary signal of unrestrained animals.

The schematic diagram of the pilot system we used to extract cardiopulmonary signal of unrestrained animals from video data is presented in Figure 2.

According to several studies [16,17,32] with human subjects, the heart and respiration muscles cause motion of the thoracoabdominal wall with amplitude variations from 0.2 mm to 12 mm at rest. These variations in the thoracoabdominal wall as a result of cardiopulmonary activity directly causes changes of reflected intensity values in video sequences. To detect these variations, a series of image-and video-processing techniques were applied to analyse and extract intensity values of interest from video data. First, the input video was converted to frame sequences. Since the camera we used captured video in RGB color space and to separate the intensity information from the color information, the RGB color space was converted to the YCbCr color space using MATLAB’s built-in command called ‘rgb2ycbcr’ as follows:

(1) $$\left[\begin{array}{c} Y\\ C_{b}\\ C_{r} \end{array}\right]=\left[\begin{array}{ccc} 65.481 & 128.553 & 24.966\\ -39.797 & -74.203 & 112\\ 112 & -93.786 & -18.2214 \end{array}\right]\left[\begin{array}{c} R\\ G\\ B \end{array}\right]+\left[\begin{array}{c} 16\\ 128\\ 128 \end{array}\right]$$

where Y is the intensity channel with a range from 16 to 235, while *C_b_* and *C_r_* have a range from 16 to 240. The second processing step was to manually localise ROIs in the thoracoabdominal region where the cardiopulmonary signal is most pronounced using MATLAB’s built-in command ‘ginput’. The ROIs were outlined by rectangles. The next processing step was to average the intensity pixel values over the image sequences of the selected ROI from the Y component of the YC_b_C_r_ color space, as follows:

(2) $$i_{Y}\left(t\right)=\frac{\sum_{x,y\in ROI}I\left(x,y,t\right)}{\left|ROI\right|}$$

where I(x,y,t) is the intensity pixel value at image location (x,y) over time (t) from recorded frames, and |ROI| is the size of the selected ROI. A wavelet signal-denoising method based on an empirical Bayesian method with a Cauchy prior was used to remove the motion artefact noise from $i_{Y}\left(t\right)$, resulting from animal movement during the recording. MATLAB’s built-in command, called ‘wdenoise’, was utilised to denoise the signal at 4 levels using wavelet Daubechies family (db15), universal threshold as denoising method and level-dependent approach as a noise estimation method at each resolution level. The process of the signal denoising was then followed by applying a moving average filter with span equal to 5 to smooth the denoised signal using MATLAB’s built-in command, named ‘smooth’. A spectral analysis method based on the fast Fourier transform (FFT) was then applied to transform the smoothed signal, $I_{Y_{smoothed}}\left(t\right)$, from the time domain to the frequency domain, followed by applying two separating ideal band-pass filters with selected frequencies according to the animal’s cardiopulmonary range (see Table 1), to separate the cardiac signal from the breathing signal. The inverse FFT was then applied to the filtered signals to obtain both the cardiac and breathing signals $I_{Y_{H}}\left(t\right)$ and $I_{Y_{B}}\left(t\right)$.

Next, a peak detection method based on the wavelet transform was carried out to identify the periodicity of peaks, their locations and number of peaks of the acquired signals. Continuous wavelet transformation (CWT) is defined as the scalar multiplication of the acquired signals, $I_{Y_{H}}\left(t\right)$ and $I_{Y_{B}}\left(t\right)$, and the scaled, shifted versions of the wavelet mother function ψ for each signal. Mathematically, the CWT functions on the signals $I_{Y_{H}}\left(t\right)$ and $I_{Y_{B}}\left(t\right)$ at point (s,h) are described as [33,34]

(3) $$W_{H_{\left(s,h\right)}}=\overset{+\infty }{\underset{-\infty }{\int }}I_{Y_{H}}\left(t\right)\;\psi_{s,h}\left(t\right)\;dt,\psi_{H_{s,h}}\left(t\right)=\frac{1}{\sqrt{s}}\psi \left(\frac{t-h}{s}\right)$$

(4) $$W_{B_{\left(s,h\right)}}=\overset{+\infty }{\underset{-\infty }{\int }}I_{Y_{B}}\left(t\right)\;\psi_{s,h}\left(t\right)\;dt,\psi_{B_{s,h}}\left(t\right)=\frac{1}{\sqrt{s}}\psi \left(\frac{t-h}{s}\right)$$

where $I_{Y_{H}}\left(t\right)$ and $I_{Y_{B}}\left(t\right)$ are the cardiac and breathing signals after denoising and smoothing, $\psi_{H_{s,h}}\left(t\right)$ is the wavelet function ψ(t) of the cardiac signal and $\psi_{B_{s,h}}\left(t\right)$ is the wavelet function ψ(t) of the breathing signal, both translated by scale s and shifted by h. The outcome of CWT coefficients contain patterns of peaks and periodicity which can be used to detect the number of peaks, their locations and their strengths in both signals. Varying scales in wavelet functions yields different width wavelets, and thus all peaks in $I_{Y_{H}}\left(t\right)$ and $I_{Y_{B}}\left(t\right)$, regardless of their width, can be detected.

Finally, to calculate PR (beats per minute b/m) and BR (respiration per minute r/m), the following equation is used:

(5) $$PR,\;BR=\frac{60pF_{r}}{n}$$

where p is the number of peaks of the acquired signal, n is the number of frames of the selected video and $F_{r}$ is the video frame rate.

### 2.4. Data MATLAB Graphical User Interface (GUI)

**Figure 3:**
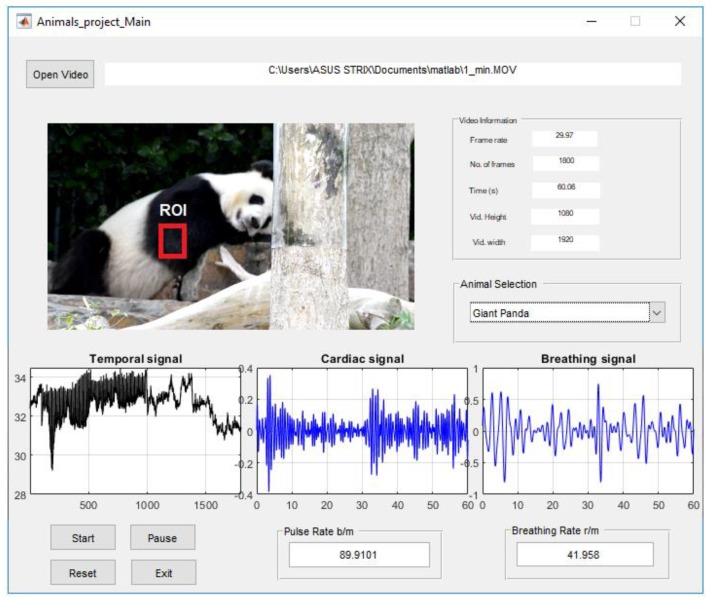
The graphical user interface (GUI) main panel of the experimental proposed image analysing system.

A GUI model was implemented in the MATLAB environment R2018b (MathWorks, NSW, Australia) with the Microsoft Windows 10 operating system to allow the user to load video data, manually select the animal according to its cardiopulmonary range, select the ROI where the cardiopulmonary signal was most apparent, and execute the algorithm. The experimental proposed graphical user interface (GUI) provides an easy tool to see video information and the selected ROI and enable the user to recognize the cardiopulmonary readings. Figure 3 shows the GUI main panel of the experimental proposed image analysing system.

The upper right of the GUI panel displays information of the input video (frame rate, number of frames, time and frame resolution). Because each animal has different frequencies of cardiopulmonary activity, a pop-up menu in the middle right of the GUI panel has been used to select the species according to the frequency of its cardiopulmonary range (see Table 1). After running the start button, a single frame appears in the top left of the GUI panel and the user can manually select the ROI where the cardiopulmonary motion is most apparent. The middle region of the GUI panel displays the cardiopulmonary signals, while two edit texts in the lower right of the GUI panel provide the BR and BR readings for the selected animal. Other buttons can be used to pause, reset or exit the algorithm.

## 3. Experimentation and Results

This section shows the feasibility of the proposed imaging system to estimate PR and BR based on motion of the body surface caused by cardiopulmonary activity without touching the animal’s body. This current study’s outcomes concerning PR and BR are in agreement with the respective normal cardiopulmonary range for the species listed in Table 1. Three time periods all of about 5 s in length were selected from the video data (video duration of 1 min) to recover the PR and BR for each animal by averaging the measurements over these periods.

**Figure 4:**
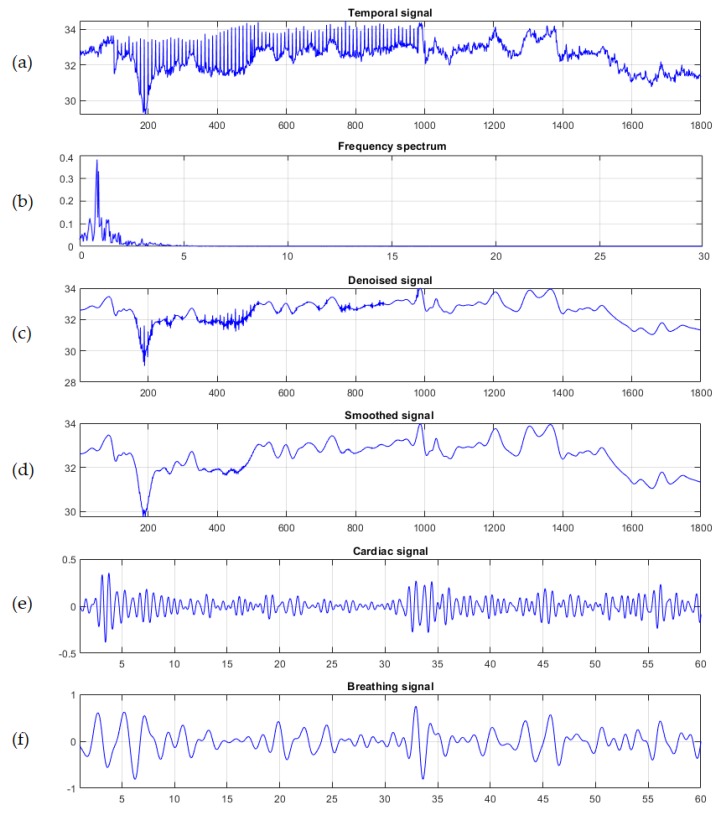
Temporal signal analysis of the giant Panda, (**a**) One minute observed time series signal $i_{Y}\left(t\right)$ of the selected region of interest (ROI), (**b**) the frequency spectrum, (**c**) the denoised signal using wavelet, (**d**) the smoothed signal using moving average filter with span equal to 5, (**e**) the cardiac signal after applying a band-pass filter of 1.1667 to 2 Hz, and (**f**) the breathing signal after applying a band-pass filter of 0.333 to 0.667 Hz.

The results from the giant panda (Ailuropoda melanoleuca) showed that motion caused by cardiopulmonary activity could be used to extract the cardiopulmonary signal on the selected ROI as shown in Figure 3. Thus, both PR and BR could be estimated by applying a spectral analysis method based on FFT on the averaged intensity signal $I_{Y_{smoothed}}\left(t\right)$. The temporal signal and frequency spectrum of the $i_{Y}\left(t\right)$ are shown in Figure 4a,b. The denoised signal using wavelet Daubechies is shown in Figure 4c and the smoothed signal using moving average filter with span equal to 5 is shown in Figure 4d. Since the Panda’s physiological parameters falls within a frequency band between 1.167 and 2 Hz for the cardiac signal and 0.333 to 0.667 Hz for the breathing signal [35], we used band-pass filters with selected frequencies of 1.17 to 2 Hz and 0.33 to 0.67 Hz, corresponding to 70–120 b/m and 20–40 r/m, respectively, followed by inverse FFT to obtain both the cardiac signal and breathing signal as shown in Figure 4e,f, respectively.

After determining the number of peaks for each signal, Equation (5) was used to calculate the PR and BR over three periods (00:15 s, 00:30 s and 00:45 s). The giant panda presented a mean PR of 87.51 ± 14 b/m and a mean BR of 43.59 ± 3 r/m as shown in Table 1, which are within the normal cardiopulmonary range for the species.

Table 1 demonstrates the normal cardiopulmonary ranges of 10 animals against the mean values of the PR and BR over three periods (00:15 s, 00:30 s and 00:45 s) obtained by the proposed imaging system.

The results from the African lions (Panthera leo) of 1 min video duration at 3 periods (00:15 s, 00:30 s and 00:45 s) also showed that the possibility of extracting the cardiopulmonary signal on the selected ROI after applying band-pass filters with selected frequencies of 0.7 to 1.27 Hz and 0.233 to 0.533 Hz, corresponding to 42–76 b/m and 14–32 r/m, respectively. The African lion (Panthera leo) presented a mean PR of 54.04 ± 11 b/m and a mean BR of 22.33 ± 4 r/m, whereas it was 55.82 ± 11 b/m and 21.92 ± 2 r/m for the lioness, which are also within the normal cardiopulmonary range.

To obtain physiological parameters from the Sumatran tiger (Panthera tigris sumatrae) over 1 min. video duration at 3 periods (00:15 s, 00:30 s and 00:45 s), we applied band-pass filters with selected frequencies of 0.933 to 1.617 Hz and 0.117 to 0.667 Hz that the cardiopulmonary signal falls within that corresponds to 56–97 b/m and 7–40 r/m. The Sumatran tiger presented mean values within the normal cardiopulmonary range of 76.67 ± 12 b/m and 21.79 ± 3 r/m for the PR and BR, respectively as shown in Table 1.

To obtain physiological parameters from the koala (Phascolarctos cinereus) over 1 min. video duration at 3 periods (00:15 s, 00:30 s and 00:45 s), we applied band-pass filters with selected frequencies of 1.083 to 1.5 Hz and 0.167 to 0.25 Hz where the cardiopulmonary signal for the species falls, corresponding to 65–90 b/m and 10–15 r/m. The Koala presented mean values within the normal cardiopulmonary range of 75.92 ± 13 b/m and 11.89 ± 1 r/m for the PR and BR, respectively, as shown in Table 1.

The PR and BR measurements for the red kangaroo (Macropus rufus) were extracted by averaging the measurements from three time periods (00:15 s, 00:30 s and 00:45 s). The results were 92.85 ± 12 b/m and 45.59 ± 3 r/m for the PR and BR, respectively after applying band-pass filters of 1.317 to 1.667 Hz for the cardiac signal and 0.433 to 0.983 Hz for the breathing signal.

The mean values of PR and BR measurements for the alpaca (Vicugna pacos) from three periods (00:15 s, 00:30 s and 00:45 s) were 68.66 ± 12 b/m and 25.65 ± 7 r/m for the PR and BR, respectively, after applying band-pass filters of 1 to 1.5 Hz for the cardiac signal and 0.167 to 0.5 Hz for the breathing signal.

The mean values of PR and BR measurements for the little blue penguin (Eudyptula minor) from three periods (00:15 s, 00:30 s and 00:45 s) were 219.29 ± 20 b/m and 11.05 ± 7 r/m for the PR and BR, respectively after applying band-pass filters of 1.883 to 4.667 Hz for the cardiac signal and 0.117 to 0.417 Hz for the breathing signal.

The mean value of PR measurements from the Sumatran orangutan (Pongo abelii) and Hamadryas baboon (Papio hamadryas) videos at three time periods were 120.61 ± 15 b/m and 119.08 ± 16 b/m, respectively after applying band-pass filters of 1.92 to 2.02 Hz (115–121 b/m) and 1.67 to 2.57 Hz (100–154 b/m), while the BR measurements were 32.89 ± 6 r/m and 30.33 ± 8 b/m after applying band-pass filters of 0.4 to 0.467 Hz (24–28 r/m) and 0.2 to 0.667 Hz (12–40 r/m), respectively.

**Figure 5:**
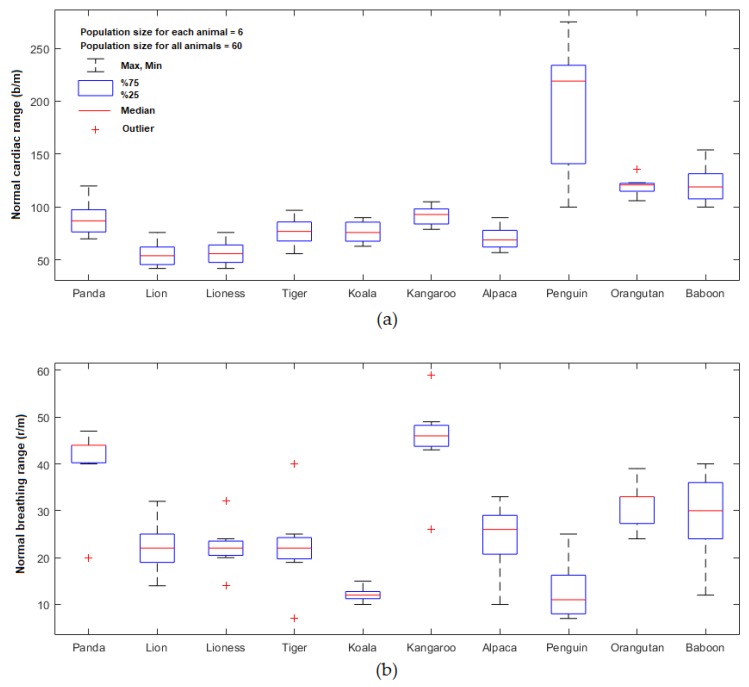
Box plots show the minimum, maximum, 25–75th percentile and median value of the cardiopulmonary range obtained from 10 zoo animals for (**a**) PR results, and (**b**) BR results.

The box plots show the distributions of six cardiopulmonary values per minute obtained from each animal (population size for all animals equals to 60) using the proposed imaging system. The box plot illustrated in Figure 5a shows the minimum and maximum values of the PR per minute and interquartile range (25–75th percentile), while the central red line shows the median value of the PR. The box plot illustrated in Figure 5b shows these values regarding the BR per minute.

Based on all the PR results obtained from all animals illustrated Figure 5a, the cardiac signals fall within a frequency range of 0.717 to 4.583 Hz (minimum and maximum value) corresponding to 43 to 275 b/m with the average median value of 87.3 b/m. The 25–75th percentile from all population sizes were 66.5 b/m and 113.5 b/m, respectively. Based on all the BR results obtained from all the animals illustrated Figure 5b, the breathing signals fall within a frequency range of 0.133 to 0.983 Hz (minimum and maximum) corresponding to 8 to 59 r/m with the average median value of 24.5 r/m. The 25–75th percentile from all population sizes were 17.25 r/m and 33.5 r/m, respectively.

## 4. Discussion

The technique presented shows promising results. All of the experimental subjects displayed motion consistent with both cardiac and respiratory activity as found in publications using traditional clinical instrumentation. There are a number of challenges that stand in the way of developing a fielded prototype.

The animal subjects tend to move during filming, where a human subject might be instructed to not move. Some of this movement might be compensated for using image-processing techniques to extract the different components of movement. Periods of low movement such as during feeding or sleep might be exploited to obtain the measurements. Such improvements would probably reduce the variations we have recorded substantially. Since the technique is non-invasive, of low energy and unobtrusive, it might be possible to achieve even more meaningful data by focusing on long-term observations. This may expose trends and achieve more stable average readings.

The question of long-term averages and unattended monitoring does raise some additional challenges. Animals are not subjects of typical mobile device applications or large consumers of the technology, and thus software that is used for recognizing parts of the human body, such as human activity vibration monitoring systems, do not exist [45]. This is particularly true for diverse exotic animals that might be found in a zoo setting. This study thus used manually selected regions of interest, relying on the human operator. The range of complexity and usability of a future veterinary instrument is wide. A fielded prototype might be expected to manage ROI selection and tracking, or even automatic management of the entire process including the selection of individual animals. Alternatively, a video camera in the field with software for analysis applied off-line might be entirely adequate.

As a pilot study the control data has been cardiac and respiratory rate averages for each species based upon the literature. The respiratory rate is obvious to the eye with many species and could be extracted by an operator as an experimental control from the video used for the computer vision solution. A controlled study is required where there is reference data provided for the cardiac rate. Ideally, synchronized video, cardiac and respiratory events would be captured in such a study.

An issue with obtaining this sort of data is that most of the animals observed are not amenable to instrumentation while conscious and the remainder may be agitated by the interaction. Generally, the animals are anaesthetized before being clinically assessed in detail. It is thus likely that the next stage of research will be challenging and will examine fewer species than this broad study. There are a number of ethical approaches to capturing this data that should provide a controlled data set while creating no additional procedures for the subjects.

Many studies [46,47,48,49,50,51] have used video magnification techniques with human subjects to extract vital signs from appropriate ROI when the cardiopulmonary activity is hard or impossible to recognize using a video camera. These techniques may provide promising results for future work directions.

## 5. Conclusions

We have presented a pilot study that has demonstrated that practical non-contact respiratory rate and cardiac rate may be measured from video imagery of diverse species of animals in a zoo setting. The technique relies on the detection of motion that appears to be visible even when the subjects are covered in thick fur or hair. Measurements made by video provided rates that were close to those documented in the literature about these species. The minimum and maximum values of the PR obtained from all animals (population size = 60 readings) were 43 and 275 b/m, respectively. The 25–75th percentile from all population sizes were 66.5 b/m and 113.5 b/m, respectively, and the average median value for the PR was 87.3 b/m which all fall within the normal cardiac frequency range of the selected animals. The minimum and maximum values of the BR obtained from all animals (population size = 60 readings) were 8 and 59 r/m, respectively. The 25–75th percentiles from all population sizes were 17.25 r/m and 33.5 r/m, respectively, and the average median value for the BR was 24.5 r/m which also falls within the normal breathing frequency range of the selected animals.

The technique holds promise as a tool for improved management of animals. Future work will focus on comparing the video technique to controlled data captured at the same time for more certainty about the value of this method. We will also explore more challenging scenarios than enclosures in zoos by considering open range settings and wild populations of animals.

## Figures and Tables

**Table 1:** A normal cardiopulmonary range of animals against those obtained by the proposed imaging system.

Animals	Normal Cardiopulmonary Range	Cardiopulmonary Signal pulse rate (PR b/m) and Breathing Rate (BR r/m) (the Average from 3 Points)
Giant panda (Ailuropoda melanoleuca)	70–120 b/m [35] 20–40 r/m	87.51± 14 b/m 43.59 ± 3 r/m
African lion (Panthera leo)	42–76 b/m [36] 14–32 r/m [37]	54.04 ± 11 b/m 22.33 ± 4 r/m
African lioness (Panthera leo)	42–76 b/m [36] 14–32 r/m [37]	55.82 ± 11 b/m 21.92 ± 2 r/m
Sumatran tiger (Panthera tigris sumatrae)	56–97 b/m [36] 7–40 r/m [38]	76.67 ± 12 b/m 21.79 ± 3 r/m
Koala (Phascolarctos cinereus)	65–90 b/m [39] 10–15 r/m	75.92 ± 13 b/m 11.89 ± 1 r/m
Red kangaroo (Macropus rufus)	79–100 b/m [40] 26–59 r/m	92.85 ± 12 b/m 45.59 ± 3 r/m
Alpaca (Vicugna pacos)	60–90 b/m [41] 10–30 r/m	68.66 ± 12 b/m 25.65 ± 7 r/m
Little blue penguin (Eudyptula minor)	113–280 b/m [42] 7–25 r/m	219.29 ± 20 b/m 11.05 ± 7 r/m
Sumatran Orangutan (Pongo abelii)	115–121 b/m [43] 24–28 r/m	120.61 ± 15 b/m 32.89 ± 6 r/m
Hamadryas baboon (Papio hamadryas)	100–154 b/m [44] 12–40 r/m	119.08 ± 16 b/m 30.33 ± 8 b/m
